# BRD4 Inhibitor Alleviates Recurrent Spontaneous Abortion via Regulating BRD4/STAT3/IL‐17A Axis to Decrease the Th17 Cell Differentiation

**DOI:** 10.1002/rmb2.12682

**Published:** 2025-10-15

**Authors:** Fen Liu, Zhenhui Zhang, Chengying Yang

**Affiliations:** ^1^ The Obstetrics and Gynecology Department The First Hospital of Changsha Changsha China

**Keywords:** BETi, BRD4, IL‐17A, recurrent spontaneous abortion, STAT3, Th17 cell differentiation

## Abstract

**Purpose:**

Recurrent spontaneous abortion (RSA) is an abnormal phenomenon that severely affects women's quality of life. Inhibiting Th17 cell differentiation can alleviate RSA. This research explored the mechanism by which BRD4 Inhibitor (BETi) suppressed the differentiation of Th17 cells to mitigate RSA.

**Methods:**

PBMCs and Naive CD4^+^ T cells were induced to differentiate into Th17 and Treg cells. An abortion‐prone pregnancy mouse model was constructed by intraperitoneal injection of lipopolysaccharide. The Th17/Treg ratio was determined by flow cytometry. The association between STAT3 and IL‐17A promoter was investigated by ChIP and dual luciferase assays. Co‐IP and yeast two‐hybrid assays were used to determine BRD4 binding to STAT3. The markers of Th17/Treg cell differentiation and lipid synthesis were checked by ELISA, IHC, RT‐qPCR, and Western blot.

**Results:**

The Th17/Treg ratio and the expression levels of BRD4, STAT3, and IL‐17A were elevated, while STAT5b expression was down‐regulated in RSA patients. BETi or STAT3 knockdown decreased the differentiation of Th17 cells and lipid synthesis. BRD4 inhibition impaired STAT3‐mediated IL‐17A transcription. BETi inhibited embryo absorption in mice.

**Conclusions:**

BETi inhibits the differentiation of Th17 cells in RSA by reducing the STAT3‐mediated IL‐17A expression.

## Graphical abstract

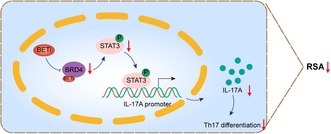

**Abbreviations**
ACC‐1: acetyl‐CoA carboxylase 1
ADP/ATP: adenosine diphosphate/Adenosine triphosphate
AMPK‐α1: AMP‐activated protein kinase alpha1
AP: abortion‐prone pregnancy
BETi: bromodomain and extraterminal domain inhibitor
BRD4: bromodomain‐containing protein 4
ChIP: chromatin immunoprecipitation
Co‐IP: co‐immunoprecipitation
DMSO: dimethyl sulfoxide
FBS: fetal bovine serum
FOXP3: forkhead box protein 3
IL‐17A: interleukin‐17A
LPS: lipopolysaccharide
NADP: nicotinamide adenine dinucleotide phosphate
NP: normal pregnancy
PBMCs: peripheral blood mononuclear cells
PBS: phosphate‐buffered saline
PD‐1: programmed cell death‐1
PMA: phorbol 12‐myristate 13‐acetate
PNS: Panax notoginseng saponins
rhCOLI: recombinant humanized type I collagen
RORγt: retinoic acid receptor‐related orphan receptor gt
RSA: recurrent spontaneous abortion
SREBP‐1c: sterol regulatory element‐binding protein‐1c
STAT3: signal transducer and activator of transcription 3
TGF‐β: transforming growth factor β
Th17: T helper type 17
Treg: regulatory T

## 1 Introduction

Recurrent spontaneous abortion (RSA) is a distressing pregnancy disorder associated with genetic, endocrine, immunologic, anatomical, and infectious factors [1]. It not only hurts women's physical and mental health but also puts a lot of pressure on marital and family relationships [2]. In recent years, several advances have been made in the therapeutic approach to RSA, such as anticoagulants combined with anti‐inflammatory drugs or the immunosuppressant Cyclosporine A alone [3, 4]. However, more research is needed to explore the pathogenesis of RSA for better therapeutic outcomes and earlier clinical applications.

It has been implicated that an imbalance in the Th17/Treg cell ratio and the enhanced differentiation capacity of Th17 cells is important contributors to the occurrence of RSA. The increased differentiation capacity of Th17 cells is an important contributor to the occurrence of RSA [5]. The T helper type 17 (Th17) cell, a subpopulation of T cells characterized by the secretion of interleukin 17 (IL‐17), has been reported to be involved in the regulation of several autoimmune diseases and inflammatory responses [6, 7], and play a crucial role especially in pregnancy [8]. For example, IL‐17 secreted by Th17 cells induced fetal loss by promoting an inflammatory response [9, 10]. Recombinant adiponectin attenuated abortion in mice by activating of the p38MAPK/STAT5 pathway to decrease the Th17 cell population and function [11]. The number of Th17 cells was increased in the peripheral blood of RSA patients [12]. Therefore, further studies are necessary to explore the molecular mechanisms inhibiting Th17 differentiation and regulating Th17/Treg homeostasis, which may provide a new theoretical basis for alleviating RSA.

Bromodomain‐containing protein 4 (BRD4), as a member of the bromodomain and extraterminal domain (BET) family [13]. It has been shown that BRD4 is required for regulating cell cycle progression, cell differentiation, and tissue formation [14]. For example, the suppression of BRD4 inhibited peripheral plasma cell differentiation to alleviate systemic lupus erythematosus [15]. BRD4 is also an important immunomodulator in regulating the immune microenvironment of tumors, autoimmune diseases, and inflammatory diseases in the immune response [16]. BET inhibitor (BETi) hinders disease progression by suppressing BRD4. For example, the selective BRD4 inhibitor of JQ1 restricted retinoblastoma cell growth by inducing cell cycle arrest and apoptosis [17]. dBET1, a newly synthesized BETi, attenuated experimental autoimmune encephalomyelitis in mice by degrading BRD4 [18]. Nonetheless, studies on the inhibition of BRD4 in RSA by BETi have not yet been reported.

Notably, inhibiting BRD4 by BETi can mediate Th17 cell function. In inflammatory bowel disease, MS402, as a BETi, significantly reduced the differentiation capacity of Th17 cells to ameliorate the inflammatory responses by suppressing BRD4, with no effect on Th1, Th2, or Treg cells [19]. It has been implicated that BRD4 acts as a transcriptional coactivator by binding acetylated lysine residues to participate in the regulation of gene transcription [20]. BETi inhibited the proliferation of lung cancer cells by decreasing the combination of BRD4 with RelA to inhibit NF‐κB transcriptional activity [21]. BRD4 interacted with TP73 to promote RCC2 transcription, which drove esophageal squamous cell carcinoma growth [22]. Targeting CDCP1 gene transcription coactivated by BRD4 and CBP/p300 in castration‐resistant prostate cancer [23]. However, whether BRD4 facilitates Th17 cell differentiation via influencing the transcriptional regulation of downstream genes remains to be further investigated.

Signal transducer and activator of transcription 3 (STAT3) has been reported to be closely associated with BRD4. For example, BRD4 mediated TGFβ1‐induced STAT3 signaling to promote liver fibrosis in mice [24]. Inhibition of BRD4 alleviated osteosarcoma progression by reducing activation of the GP130/STAT3 signaling pathway [25]. Furthermore, STAT3 is strongly associated with miscarriage. For example, STAT3 was activated and its expression was enhanced in RSA patients [26]. SEMA4A promoted migration and proliferation of trophoblast cells by activating the STAT3, thereby improving missed abortion [27]. Herein, we identified a potential interaction between BRD4 and STAT3 by BioGRID database. However, it is not clear whether BRD4 interacts with STAT3 under RSA conditions. STAT3 has important roles in cell proliferation, differentiation, apoptosis, immune, and inflammatory responses, and is particularly critical for Th17 cell differentiation [28, 29]. It has been demonstrated that PKM2 promotes Th17 cell differentiation and autoimmune inflammation by interacting with STAT3 and enhancing its activity to increase IL‐17A expression [30]. PNS attenuated Th17 cell differentiation and inflammation in mice with collagen‐induced arthritis by inhibiting PKM2‐mediated STAT3 phosphorylation and activation to reduce IL‐17A levels [31]. Although the role of STAT3 in Th17 cell differentiation has been well reported [32, 33], the specific molecular mechanism by which STAT3 regulates IL‐17A to affect Th17 cell differentiation remains to be further confirmed. Interestingly, we previously predicted the binding sites of STAT3 to the IL‐17A promoter using the JASPAR database. As a result, BRD4 may affect Th17 cell differentiation to exacerbate RSA by regulating the process that STAT3 regulates the transcription of IL‐17A.

In summary, we proposed that BRD4 affected Th17 cell differentiation to aggravate RSA by modulating the transcriptional effects of STAT3 on IL‐17A. These findings revealed the action of BETi on RSA and its molecular mechanism and provided an important reference for subsequent clinical relief of RSA.

## 2 Materials and Methods

### 2.1 Study Population

Fifteen patients with two or more consecutive RSA were included in this study. The inclusion criteria were as follows: at least two previous spontaneous abortions, uncovered biochemical pregnancy, regular menstruation, spontaneous conception, and no history of intake of steroid hormones and toxin or drug exposure during early pregnancy. Exclusion criteria included endocrine and anatomical disorders, genetic abnormalities, and infections. Fifteen volunteers with normal pregnancy (NP) who underwent routine physical examination in the hospital simultaneously and had similar gestational weeks were selected as the experimental control group. Peripheral blood was collected from the above NP participants and RSA patients during pregnancy for isolation of peripheral cellular blood mononuclear cells (PBMCs). Early decidual tissues were gathered in 10 cases each from NP participants and RSA patients. The clinical characteristics of the studied population are summarized in Table S1. Informed consent was signed by both patients and volunteers before the start of the study. This study was approved by the Medical Ethics Committee of The First Hospital of Changsha.

### 2.2 Differentiation of Th17 Cells in Mouse

All animal experiments were conducted in accordance with the protocol approved by the First Hospital of Changsha Ethics Committee. C57BL/6N mice (8–10 weeks, 25–30 g) were purchased from Hunan Medical Laboratory Animal Center (Hunan, China). Cells were collected from the spleens of mice and prepared into single‐cell suspensions. CD4^+^ T cells were purified using the CD4^+^ T cell isolation kit (Biolegend, 480006, USA). The 96‐well plates were covered with anti‐CD3 (Elabscience, E‐AB‐F1013C, China) and incubated at 4°C overnight. Then, cell suspension, CD28 (Elabscience, E‐AB‐F1026UD, China) antibody, IL‐6 (25 ng/mL, TEASEN, 90146ES10, China), and TGF‐β (0.5 ng/mL, TEASEN, 91701ES10, China) were added to induce Th17 differentiation [30]. After 72 h of incubation, the cell stimulation cocktail (2 μg/mL) was added and incubated for 16 h. The cells were collected and assayed by a flow cytometer (BD Biosciences, CA, USA).

### 2.3 Flow Cytometry

To assess the percentage of Th17 cells, the peripheral blood was taken for isolation of PBMCs, followed by the addition of 10% FBS (S711‐001S, NSERA, Uruguay), 10 ng/mL PMA (Multi sciences, CS0001, China), and 0.5 μM ionomycin (Multi sciences, CS0002, China). Then the induced PBMCs or CD4^+^ cells were incubated with FITC‐anti‐human/mouse CD4 (BIO legend, 357405/130308, USA) and PerCP/Cyanine5.5‐labeled anti‐human/mouse IL‐17A (BIO legend, 130308/506919, USA), at 4°C for 15 min. In addition, to measure the percentage of Treg cells, samples were incubated with FITC‐anti‐human CD4 and PerCP/Cyanine5.5‐anti‐human CD25 (BIO legend, 385221, USA) at 4°C for 30 min. Cells were permeabilized and incubated with APC‐anti‐human‐FOXP3 antibody (Invitrogen, 77‐5774‐40, USA). Then the cells were resuspended in FACS solution and then detected using a flow cytometer (BD Biosciences, CA, USA). Data were analyzed using FlowJo software. The gating strategies were performed as follows: For Th17 cell detection, CD4^+^ cells were gated, and analyzed IL‐17A^+^ cells were analyzed in a CD4^+^ gate. For Treg cell detection, CD4^+^ cells were gated, and analyzed CD25^+^FOXP3^+^ cells were analyzed in a CD4 gate. Isotype (ISO) controls were set for excluding antibody nonspecific binding.

### 2.4 Cell Transfection and Treatment

Transfection was performed after the cell density reached 80%, and sh‐STAT3 and oe‐STAT3 plasmids (GenePharma, Shanghai, China) were introduced into the cells using lipo 2000 (11668030, Invitrogen, USA). The cell culture medium was OPTIM‐MEM (2537156, Gibco, USA), and cells were cultured in the incubator for 6–8 h. Subsequently, cells were cultured in a standard normal medium for 48 h, and BETi (MS402, 500 nM) was added simultaneously.

### 2.5 Construction of BRD4 Deletion Mutants With Different Structural Domains

BRD4 was truncated into three fragments: BRD4 1# (ΔBD1 truncation mutant plasmid), BRD4 2# (ΔBD2 truncation mutant plasmid), and BRD4 3# (ΔCTD truncation mutant plasmid). Subsequently, BRD4 wild‐type or different BRD4 truncation mutants were transfected into 293 T cells and identified by Western blot.

### 2.6 ELISA

Serum levels of IL‐17A were detected according to the instructions of the IL‐17A ELISA kit (Dayou, 1111702, China). Standards and samples were added to reaction wells pre‐coated with IL‐17A antibody. The OD values of the wells were measured at 450 nm using an enzyme marker (MRX II, Dynex, Chantilly, VA, USA).

### 2.7 RT‐qPCR

Cells were lysed using Trizol and then RNA was extracted using chloroform and isopropanol. Subsequently, cDNA was reverse transcribed from RNA using the PrimeScript RT reagent Kit (Takara, RR037A, Japan). According to the TB Green Premix Ex Taq kit (Takara, RR420A, Japan) instructions, we set up the PCR reaction system. PCR reactions were immediately performed on a real‐time fluorescent quantitative PCR instrument (LightCycler96, Roche, Germany). The ct values were calculated by the 2^−ΔΔCt^ method to obtain gene expression. The sequences of the qPCR primers are shown in Table 1.

**TABLE 1:** Primer sequences for qPCR.

Gene	Forward (5′–3′)	Reverse (5′–3′)
Human IL‐17A	GCCCAAATTCTGAGGACAAG	GGGGACAGTTCATGTGGT
Human STAT3	TAGCCGCTTCCTGCAAGAGT	ACAATCCGGGCAATCTCCAT
Human BRD4	GTGGTGCACATCATCCAGTC	CCGACTCTGAGGACGAGAAG
Human STAT5b	GTGTGAGAAGTTGGCCGAGA	GTGGCTGCAAACTTGGTCTG
Human GAPDH	AGGTCGGAGTCAACGGATTT	TGACGGTGCCATGGAATTTG
Mouse IL‐17A	TCTTTAACTCCCTTGGCGCA	GGACCAGGATCTCTTGCTGG
Mouse STAT3	CAATACCATTGACCTGCCGAT	GAGCGACTCAAACTGCCCT
Mouse BRD4	TTCAGCACCTCACTTCGACC	TTCAGCACCTCACTTCGACC
Mouse RORγt	TCCACTACGGGGTTATCACCT	AGTAGGCCACATTACACTGCT
Mouse GAPDH	AGGTCGGTGTGAACGGATTTG	GGGGTCGTTGATGGCAACA

### 2.8 Immunohistochemistry (IHC)

Early decidual tissues of NP participants and RSA patients were paraffin‐sectioned. The sections were dewaxed and hydrated using xylene and ethanol, respectively. They were then incubated in a 3% methanol‐H_2_O_2_ solution for antigen repair. After blocking with 5% BSA, primary antibodies such as STAT3 (1:500, 9139, CST, USA), STAT5b (1:1000, ZRB2152, Sigma‐Aldrich, USA), and BRD4 (1:20 000, 13440, CST, USA) were added for incubation, followed by secondary antibodies. Color development was then performed using DAB and observed under a microscope (Olympus, Tokyo, Japan). Finally, plates were blocked using the neutral dendrimer. The immunohistochemical results were interpreted under double‐blind conditions.

### 2.9 Western Blot

Samples to be tested were lysed on ice for 10 min using RIPA protein lysis solution (Biotech, P0013B, China) containing protease inhibitors (Biotech, P1006, China). Protein concentration was subsequently determined using the BCA kit (Biotech, P0011, China). 30 μg of total protein was thoroughly mixed with 5 × SDS uploading buffer and added to an SDS‐PAGE gel for electrophoretic separation, and then transferred to a PVDF membrane by the Trans‐Blot Turbo system. The PVDF membrane was closed with 5% skimmed milk powder at room temperature for 2 h. Primary antibodies were hybridized at 4°C overnight, such as IL‐17A (1:1000, 13838, CST, USA), RORγt (1:500, PA5‐86733, Thermo Fisher Scientific, USA), STAT3 (1:1000, 9139, CST, USA), p‐STAT3 (1:1000, 9131S, CST, USA), BRD4 (1:1000, 54615, CST, USA), p‐AMPK‐α1 (1:1000, 2537, CST, USA), AMPK‐α1 (1:1000, 2532, CST, USA), SREBP‐1c (1:500, ab28481, Abcam, USA), and ACC‐1 (1:10 000, 67373‐1‐Ig, proteintech, China). The HRP‐conjugated anti‐rabbit (1:5000, 7074S, CST, USA) or anti‐mouse (1:2000, 7076S, CST, USA) IgG was then introduced and maintained at 37°C for 1 h. Gel imager imaging was performed after incubation using an ECL chromogenic solution (Biotech, P0018S, China). Gray scale values were analyzed using ImageJ (National Institutes of Health, USA).

### 2.10 ADP/ATP and NADP/NADPH Ratio

The ADP/ATP and NADP/NADPH ratios were determined according to the instructions of the ADP/ATP Assay Kit (Sigma‐Aldrich, MAK135, USA) and the NADP/NADPH kit (Biotech, S0179, China), respectively. The reaction mixture was added for 30 min, and then the luminescence readings were detected using an enzyme marker (MRX II, Dynex, USA) to calculate the ADP/ATP ratio. Besides, cells were lysed by adding 200 μL of NADP/NADPH extract to break down the NADP. Then, 100 μL of G6PDH working solution and 10 μL of the developer were incorporated. Absorbance was read at 450 nm to calculate the NADP/NADPH ratio.

### 2.11 Chromatin Immunoprecipitation (ChIP)

The ChIP kit (Thermo Scientific, 26157, USA) was operated according to the instructions. Cell cross‐linking was performed using 1% formaldehyde and then lysed by adding cell lysis solution. Chromatin was fragmented by sonication into small fragments of 200–1000 bp, which were then incubated with Protein A/G beads, STAT3 antibody (1:100, 9139, CST, USA), and the control lgG for immunoprecipitation reactions to form Protein A/G bead‐STAT3‐DNA complexes. Subsequently, the STAT3‐DNA complexes were eluted from the magnetic beads and de‐crosslinked. Finally, the DNA was purified and then assayed by qPCR to analyze the binding relationship between IL‐17A and STAT3.

### 2.12 Dual Luciferase Assay

The binding sites between STAT3 and IL‐17A promoter were predicted by the JASPAR database. The WT or MUT gene sequences of the binding site were constructed from the prediction results and cloned into the pmirGLO vector (Youbia, VT1439, China). At the same time, the vector contains Firefly luciferase and Renilla luciferase. Subsequently, these constructed vectors were co‐transfected with the control vector or STAT3 into cells and treated with BETi. Next, dual luciferase activity was assessed by a dual luciferase reporter gene kit (Biotech, RG027, China) to compare the luciferase activity between the groups.

### 2.13 Co‐Immunoprecipitation (Co‐IP)

Cells were lysed using cell lysis solution supplemented with protease inhibitors. A diluted BRD4 antibody was added to cell lysates with pre‐washed magnetic beads and incubated overnight to form a co‐precipitated magnetic bead‐BRD4‐protein complex. The magnetic beads were then separated from the BRD4‐protein complex by elution. Finally, the protein complexes obtained were examined by Western blot to analyze the binding relationship between BRD4 and STAT3.

### 2.14 Yeast Two‐Hybrid Assay

The CTD structural domain, BD1 structural domain, and BD2 structural domain, which are missing in BRD4, were compared with full‐length wild‐type BRD4, respectively. A yeast two‐hybrid assay was performed to detect the binding structural domain of BRD4 to STAT3. The yeast two‐hybrid screen followed the matchmaker gold yeast two‐hybrid system (Takara, 630489, Japan) protocol. The STAT3 gene fragment was cloned into the pGBKT7 vector to express the fusion protein with the GAL4 DNA binding domain, and the cDNA of the BRD4 gene was cloned into the pGADT7 vector to express the fusion protein with the GAL4 activation domain. Subsequently, the fusion protein was introduced into the yeast receptor cells and co‐transformed by adding the pre‐formulated DNA transformation mix. Strains AH109 ( *Saccharomyces cerevisiae* ) were cultured in a yeast synthetic medium lacking tryptophan/leucine. Colony growth was observed after 3 days. If the yeast receptor cells can grow normally, there is a strong interaction between BRD4 and STAT3.

### 2.15 Animal Model Construction and Treatment

Female C57BL/6N mice and male BALB/C mice were mated at 8 weeks of age. The mated mice were injected intraperitoneally with 0.25 mg/kg of lipopolysaccharide (LPS) solution to construct the abortion‐prone pregnancy (AP) model [34]. At the same time, 10 mg/kg of MS402 was injected intraperitoneally twice a week for 3 weeks, and the control group was injected with saline [19]. The mice were euthanized at the end of the experiment, and the mouse uteroplacental complexes and peripheral blood were collected for further analysis. The embryo resorption rate was determined for each mouse to assess pregnancy outcome and was defined as the number of resorbed embryos/(number of resorbed embryos + number of surviving fetuses) × 100%. The animal experiment protocols involved were approved by the Animal Ethics Committee of the First Hospital of Changsha.

### 2.16 Statistical Analysis

The data were analyzed by GraphPad Prism 9.0 software. A T‐test was used to compare the differences between the two groups. Multiple comparisons were performed using one‐way analysis of variance (ANOVA) followed by Tukey's post hoc test. *p* < 0.05 was deemed to indicate a statistically significant result. The results were expressed as mean ± standard deviation (using mean ± SD).

## 3 Results

### 3.1 Th17 and Treg Cell Content Differences in Peripheral Blood of NP Participants and RSA Patients

**FIGURE 1:**
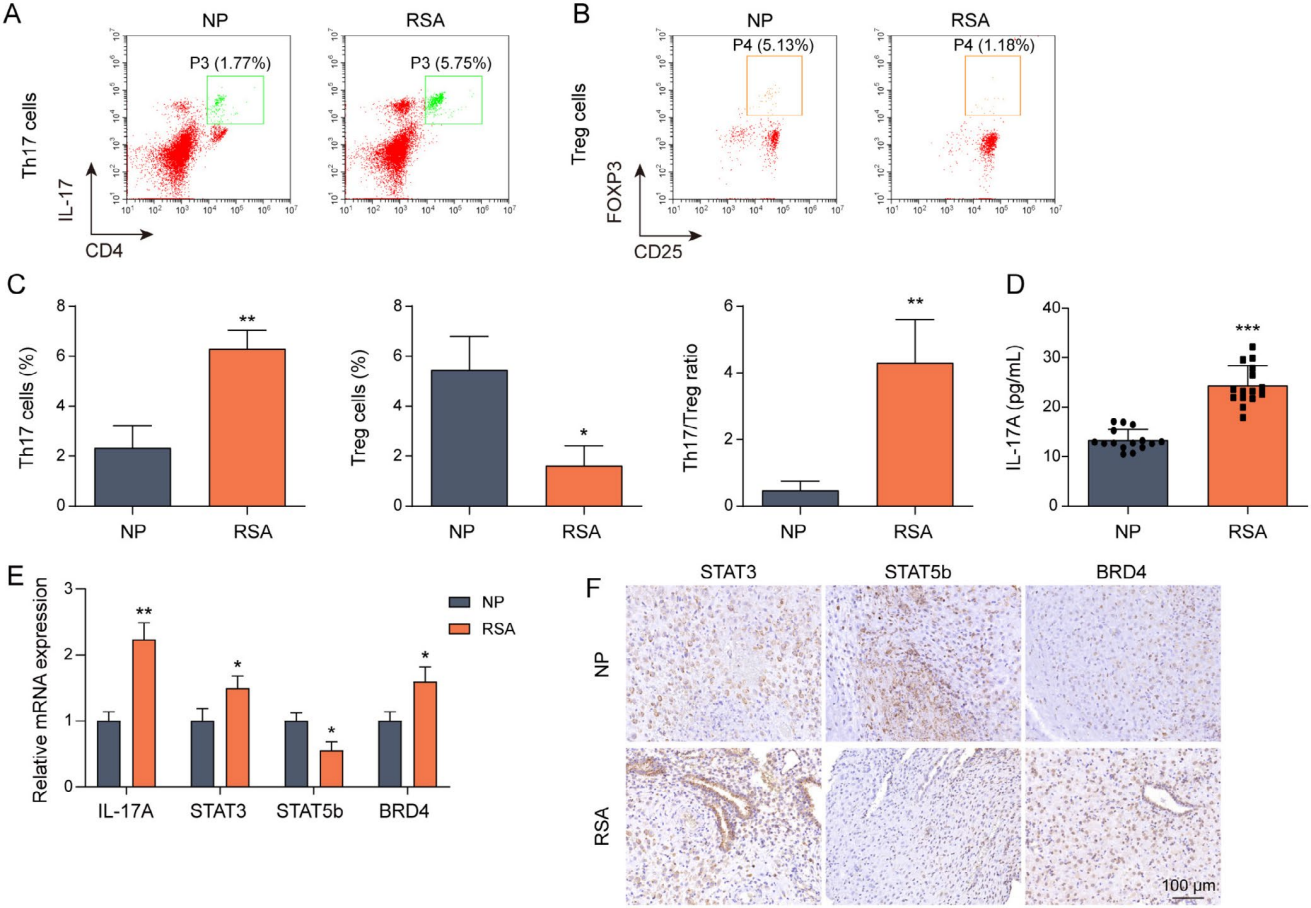
Th17 and Treg cell content differences in peripheral blood of NP participants and RSA patients. Peripheral blood from fifteen each of NP participants and RSA patients was extracted, and PBMCs were isolated, followed by 10 ng/mL PMA and 0.5 μM ionomycin to induce differentiation into Th17 cells. (A, B) Flow cytometry was used to analyze Th17 and Treg cell counts. (C) Th17/Treg ratios were calculated by flow cytometry results. (D) The level of IL‐17A was detected using ELISA. (E) The mRNA expressions of IL‐17A, STAT3, STAT5b, and BRD4 were examined by RT‐qPCR. Early decidual tissues (*n* = 10) were extracted from NP participants and RSA patients for paraffin sectioning. (F) IHC was performed to detect the protein levels of STAT3, STAT5b, and BRD4 in the tissues. *p < 0.05, **p < 0.01, ***p < 0.001.

The Th17/Treg balance has been reported to have an important protective role in RSA [5, 35]. To explore the mechanism of Th17/Treg in RSA, we collected peripheral blood from NP participants and RSA patients to isolate PBMCs, which were induced to differentiate into Th17 and Treg cells. In this study, the percentages of CD4^+^ IL‐17^+^ and CD25^+^ CD4^+^ FOXP3^+^ cells in NP participants and RSA patients were examined by flow cytometry. The results showed that the Th17 percentage was increased, the Treg percentage was decreased, and the Th17/Treg ratio was enlarged (Figure 1A–C) in RSA patients relative to NP participants. In addition, it was found that IL‐17A levels were elevated in the peripheral blood of RSA patients (Figure 1D). Meanwhile, STAT3 (Th17 differentiation transcription factor) and BRD4 expressions were up‐regulated, while STAT5b (Treg differentiation transcription factor) expression was reduced in RSA patients (Figure 1E). Finally, immunohistochemistry revealed that the expressions of STAT3 and BRD4 were increased, and the expression of STAT5b was down‐regulated in the early decidual tissues of RSA patients (Figure 1F). The above data illustrated that the balance of Th17/Treg was strongly correlated with RSA progression.

### 3.2 BRD4‐Targeted Inhibitor (BETi) Suppressed Th17 Cell Differentiation

**FIGURE 2:**
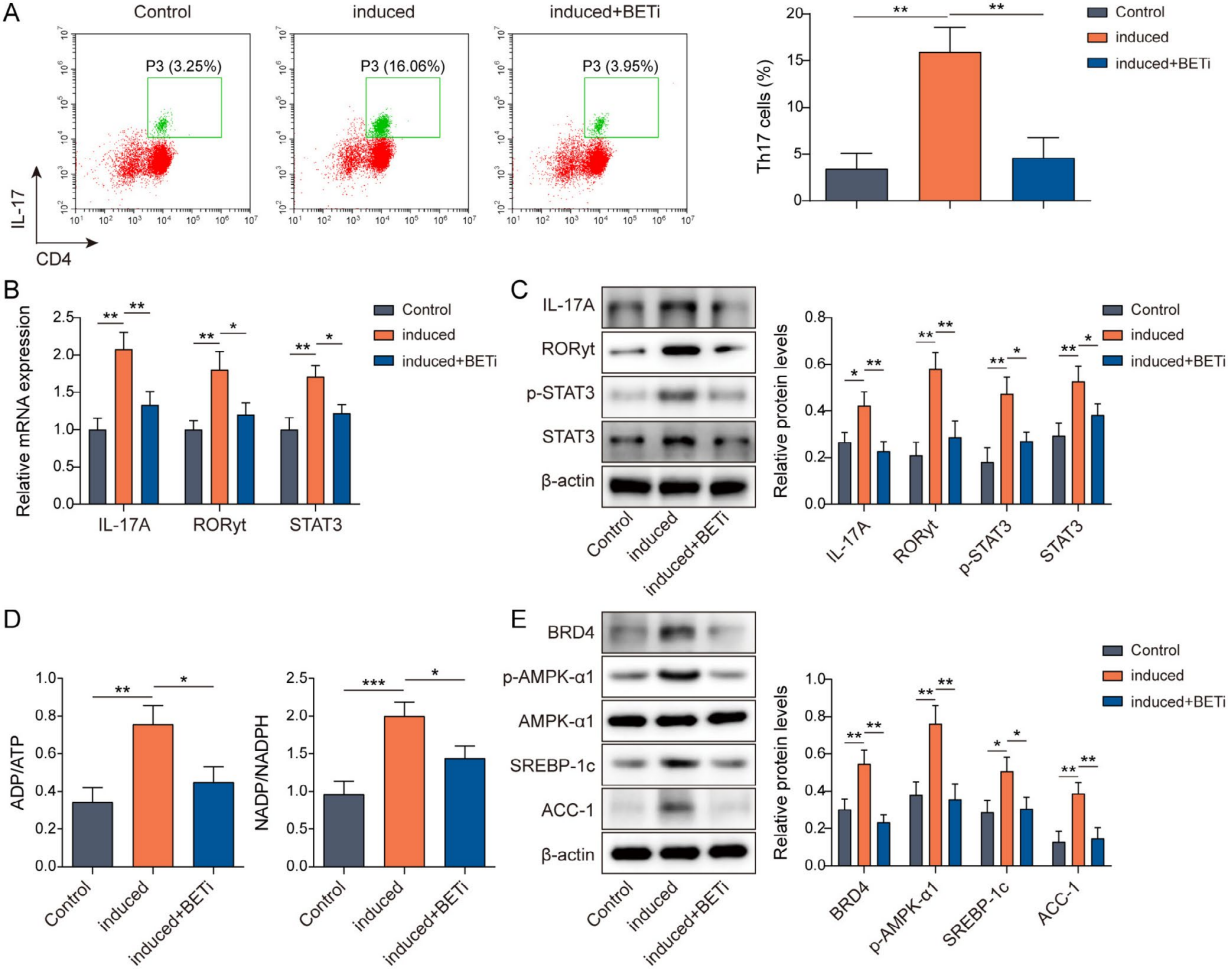
BRD4‐targeted inhibitor (BETi) suppressed Th17 cell differentiation. Naive CD4^+^ T cells were isolated from the spleens of C57BL/6N mice and subsequently induced to differentiate into Th17 cells. (A) Th17 cells were analyzed by flow cytometry. (B) RT‐qPCR was used to detect IL‐17A, RORγt, and STAT3 mRNA expression. (C) Protein levels of IL‐17A, RORγt, and STAT3 were checked by western blot. (D) ADP/ATP and NADP/NADPH ratios were examined by commercial kits. (E) Western blot was employed to analyze BRD4, p‐AMPK‐α1, AMPK‐α1, SREBP‐1c, and ACC‐1 expressions. *n* = 3. *p < 0.05, **p < 0.01, ***p < 0.001.

Subsequently, this study further explored the role of BRD4 on Th17 cell differentiation. Naive CD4^+^ T cells were isolated from the spleens of C57BL/6N mice. Next, the cells were subjected to Th17 cell differentiation and incubated with MS402 (BETi). As demonstrated in Figure 2A–C, the proportion of Th17 cells and the expression of Th17 cell differentiation‐related factors (IL‐17A, RORγt, and STAT3) were increased after the induction of differentiation, which were weakened by the addition of BETi. Moreover, the energy required for Th17 cell differentiation is mainly provided by cellular metabolism such as glycolysis, lipid synthesis, and amino acid metabolism, as reported in the literature [36]. Therefore, we found that both ADP/ATP and NADP/NADPH ratios (Figure 2D), as well as the expression of BRD4 and lipid synthesis‐related proteins (p‐AMPK‐α1, SREBP‐1c, and ACC‐1) (Figure 2E) were increased after the induction of differentiation. However, BETi treatment decreased the above ratios and the protein levels of related proteins. As a result, BETi was able to inhibit Th17 cell differentiation.

### 3.3 STAT3 Upregulated the Transcriptional Expression of IL‐17A

**FIGURE 3:**
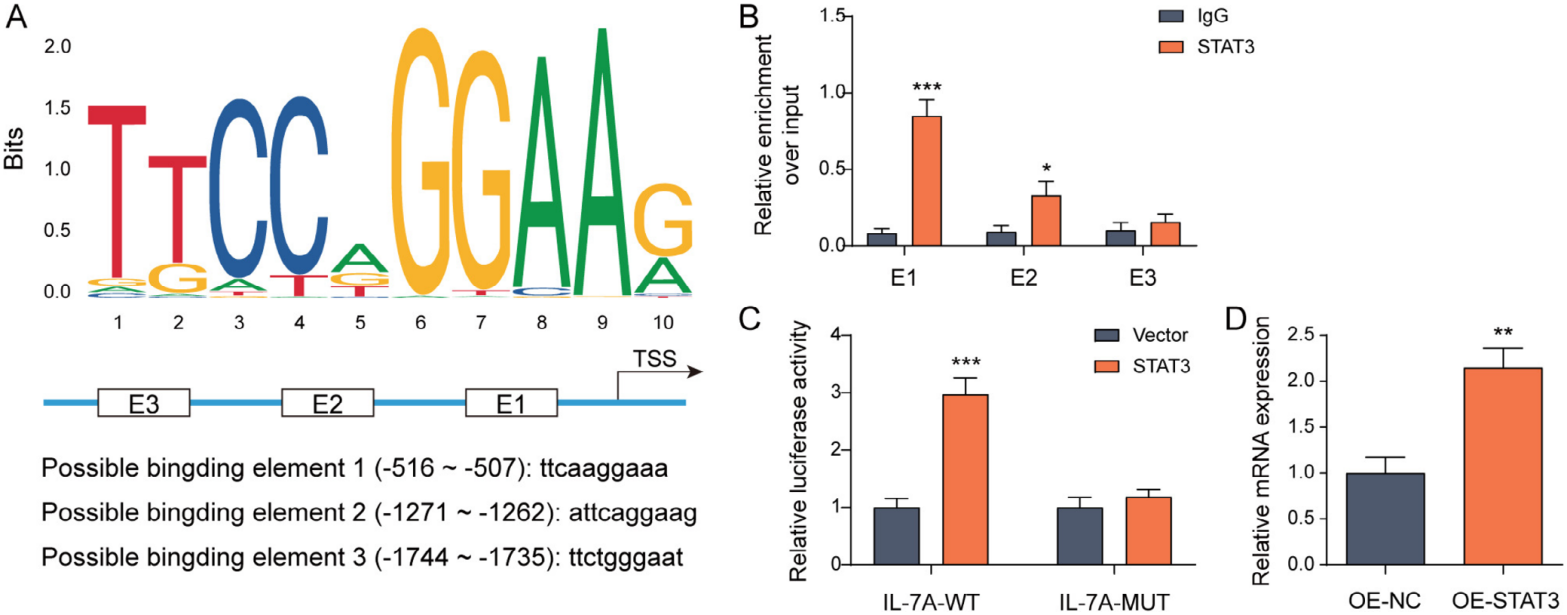
STAT3 upregulated the transcriptional expression of IL‐17A. (A) JASPAR was used to analyze the binding site between STAT3 and the IL‐17A promoter. Th17‐induced differentiation was performed in naive CD4+ T cells isolated from mouse spleens. (B) The binding association between STAT3 and the IL‐17A promoter was validated using ChIP. (C) The dual luciferase assay was employed to substantiate the combination of STAT3 with the IL‐17A promoter. (D) A cellular model of STAT3 overexpression was constructed, and the expression of IL‐17A was examined by RT‐qPCR. *n* = 3. *p < 0.05, **p < 0.01, ***p < 0.001.

Next, we investigated the mechanism by which STAT3 regulated IL‐17A. Firstly, JASPAR prediction showed that STAT3 and the IL‐17A promoter region had binding sites (Figure 3A). Besides, further validation by ChIP revealed that STAT3 could significantly enrich IL‐17A at sites E1 and E2, with E1 having a highly significant difference (Figure 3B). Meanwhile, the binding relationship between STAT3 and IL‐17A at E1 was confirmed by a dual luciferase assay (Figure 3C). Lastly, IL‐17A expression was enhanced after the overexpression of STAT3 (Figure 3D). The above data suggested that STAT3 could promote IL‐17A transcription.

### 3.4 Inhibition of STAT3 Prevented Th17 Cell Differentiation

**FIGURE 4:**
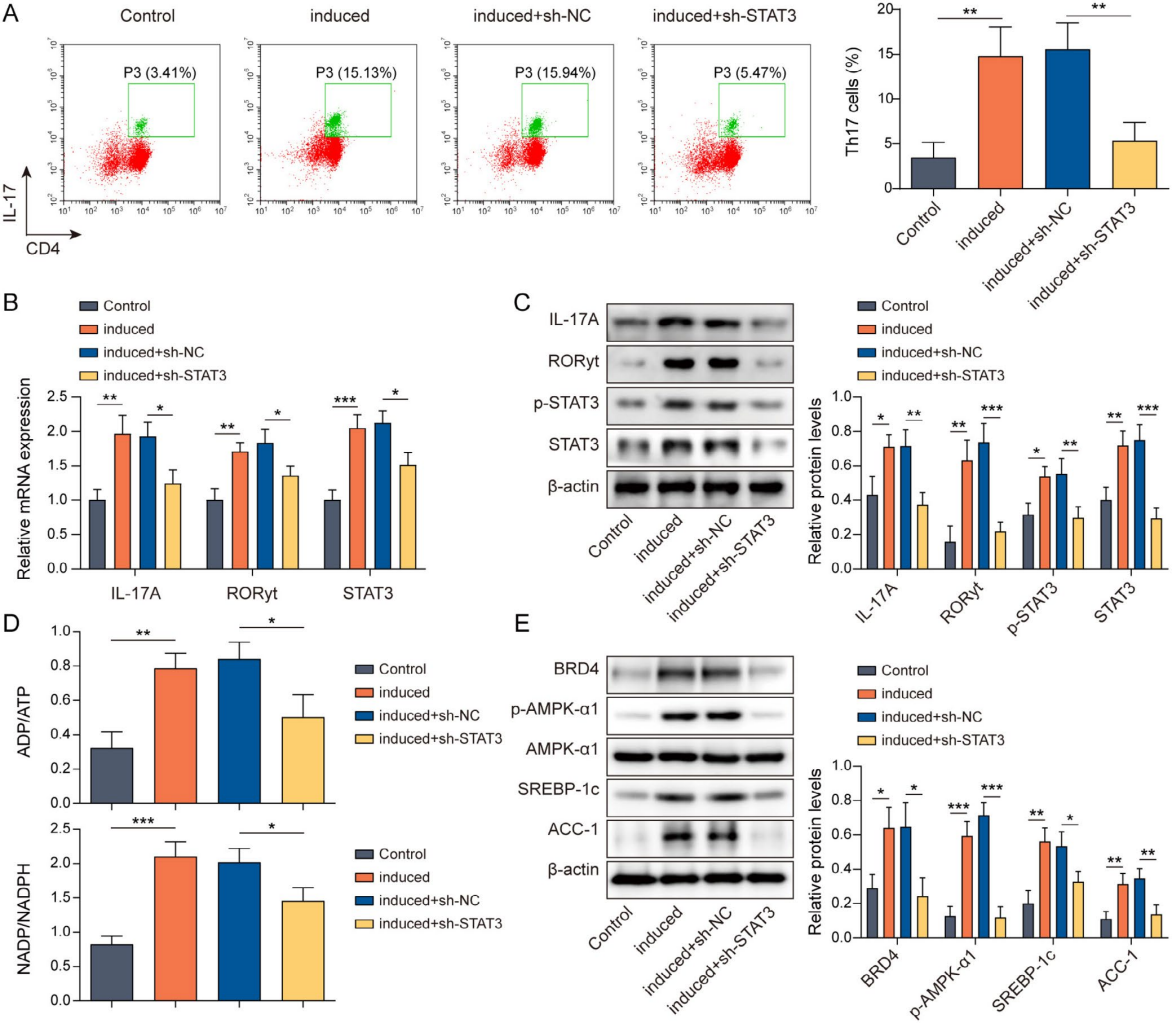
Knockdown of STAT3 prevented Th17 differentiation. Th17‐induced differentiation was performed in isolated naive CD4+ T cells from mouse spleens, followed by knockdown of STAT3. (A) Flow cytometry was used to analyze Th17 cells. (B) The expressions of IL‐17A, RORγt, and STAT3 were examined by RT‐qPCR. (C) Western blot was used to assay the IL‐17A, RORγt, STAT3, and p‐STAT3 protein levels. (D) ADP/ATP and NADP/NADPH ratios were measured using commercial kits. (E) The expressions of BRD4, p‐AMPK‐α1, AMPK‐α1, SREBP‐1c, and ACC‐1 were tested by western blot. *n* = 3. *p < 0.05, **p < 0.01, ***p < 0.001.

Subsequently, to further investigate the role of STAT3 in Th17 cell differentiation, we knocked down STAT3. The results showed that the percentage of Th17 cells (Figure 4A) and the expression of Th17 cell differentiation‐related factors (IL‐17A, RORγt, and STAT3) (Figure 4B,C) were reduced after the knockdown of STAT3. Moreover, the ADP/ATP ratio, NADP/NADPH ratio (Figure 4D), BRD4, and the markers of lipid synthesis (p‐AMPK‐α1, SREBP‐1c, and ACC‐1) levels were lowered (Figure 4E) by STAT3 knockdown. This indicated that STAT3 promoted the differentiation of Th17 cells.

### 3.5 BRD4 Bound STAT3 to Promote IL‐17A Transcription

**FIGURE 5:**
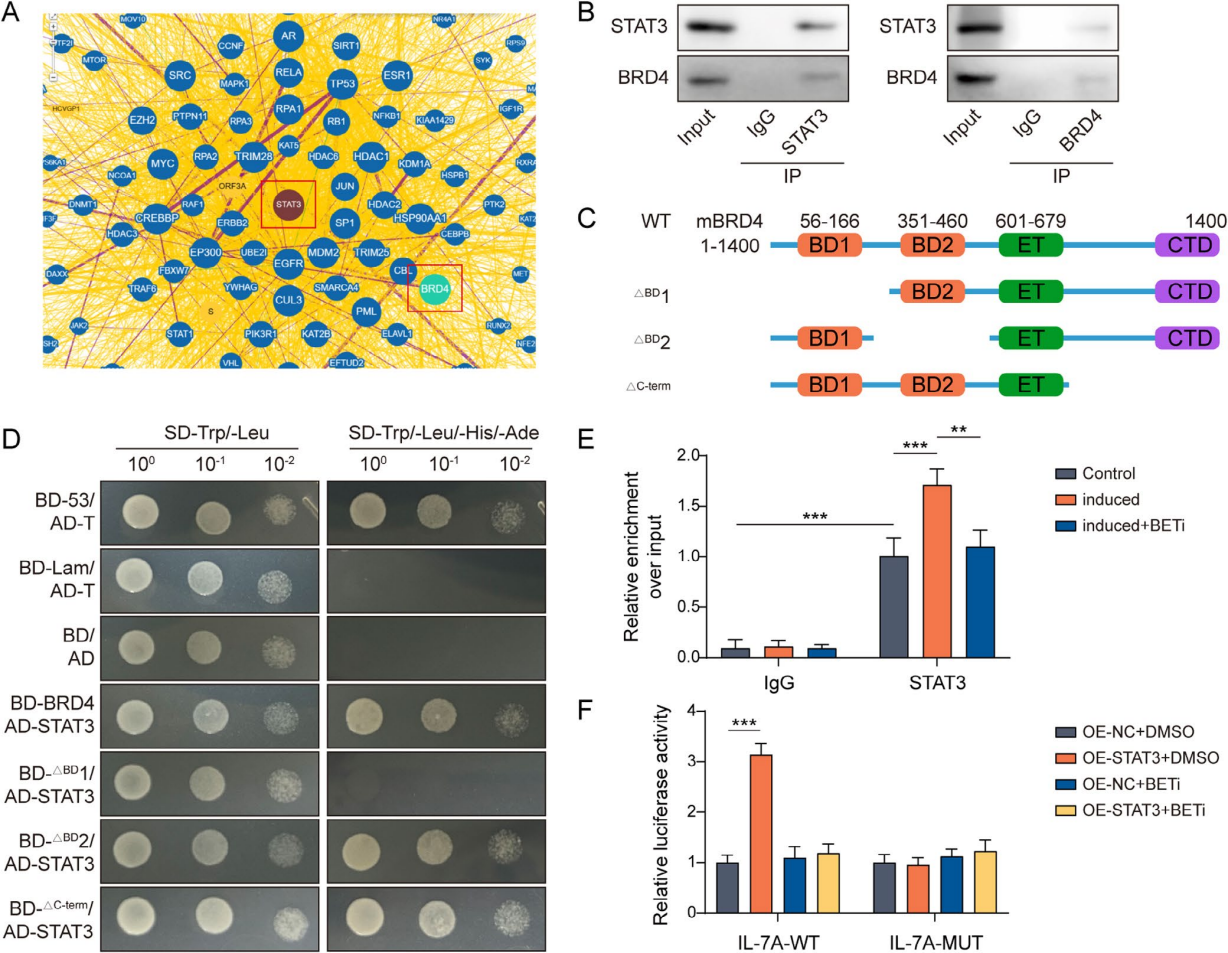
BRD4 bound STAT3 to promote IL‐17A transcription. (A) The BioGRID database was used to predict the potential binding relationship between BRD4 and STAT3. Th17‐induced differentiation was performed in naive CD4+ T cells isolated from mouse spleens. (B) BRD4 association with STAT3 binding was examined using Co‐IP. (C, D) The CTD, BD1, and BD2 structural domains of BRD4 were respectively deleted, and the binding of the BRD4 structural domain to STAT3 was detected by yeast two‐hybrid assay. BETi was added to Th17 cells. (E) ChIP was used to analyze the binding level of the IL‐17A gene promoter region to STAT3. 293 T cells were selected to construct a cell model of STAT3 overexpression, and BETi was applied. (F) The combination of STAT3 with the IL‐17A promoter was examined by the dual luciferase assay. *n* = 3. *p < 0.05, **p < 0.01, ***p < 0.001.

We then explored the effect of BRD4 on the relationship between STAT3 and IL‐17A. Prediction by the BioGRID database showed that BRD4 has a potential binding relationship with STAT3 (Figure 5A). Subsequently, Co‐IP further confirmed BRD4 interactions with STAT3 (Figure 5B). To identify the structural domains of BRD4 that interact with STAT3, the deletion of the CTD, BD1, and BD2 structural domains of BRD4 was respectively performed in this study (Figure 5C). The yeast two‐hybrid assay revealed that BRD4 interacted with STAT3 through the BD1 structural domain (Figure 5D). Furthermore, it was important that the STAT3 and IL‐17A interaction was enhanced after the induction of Th17 cell differentiation, whereas STAT3 and IL‐17A binding was reduced by adding BETi (Figure 5E). Meanwhile, STAT3 and IL‐17A binding was increased after overexpression of STAT3, while BETi treatment caused the disappearance of the STAT3 and IL‐17A interaction (Figure 5F). Altogether, the results of the above data indicated that BETi decreased Th17 cell differentiation by inhibiting STAT3‐regulated IL‐17A transcription.

### 3.6 BETi Inhibited Embryo Absorption in Miscarried Mice by Decreasing Th17 Cell Differentiation

**FIGURE 6:**
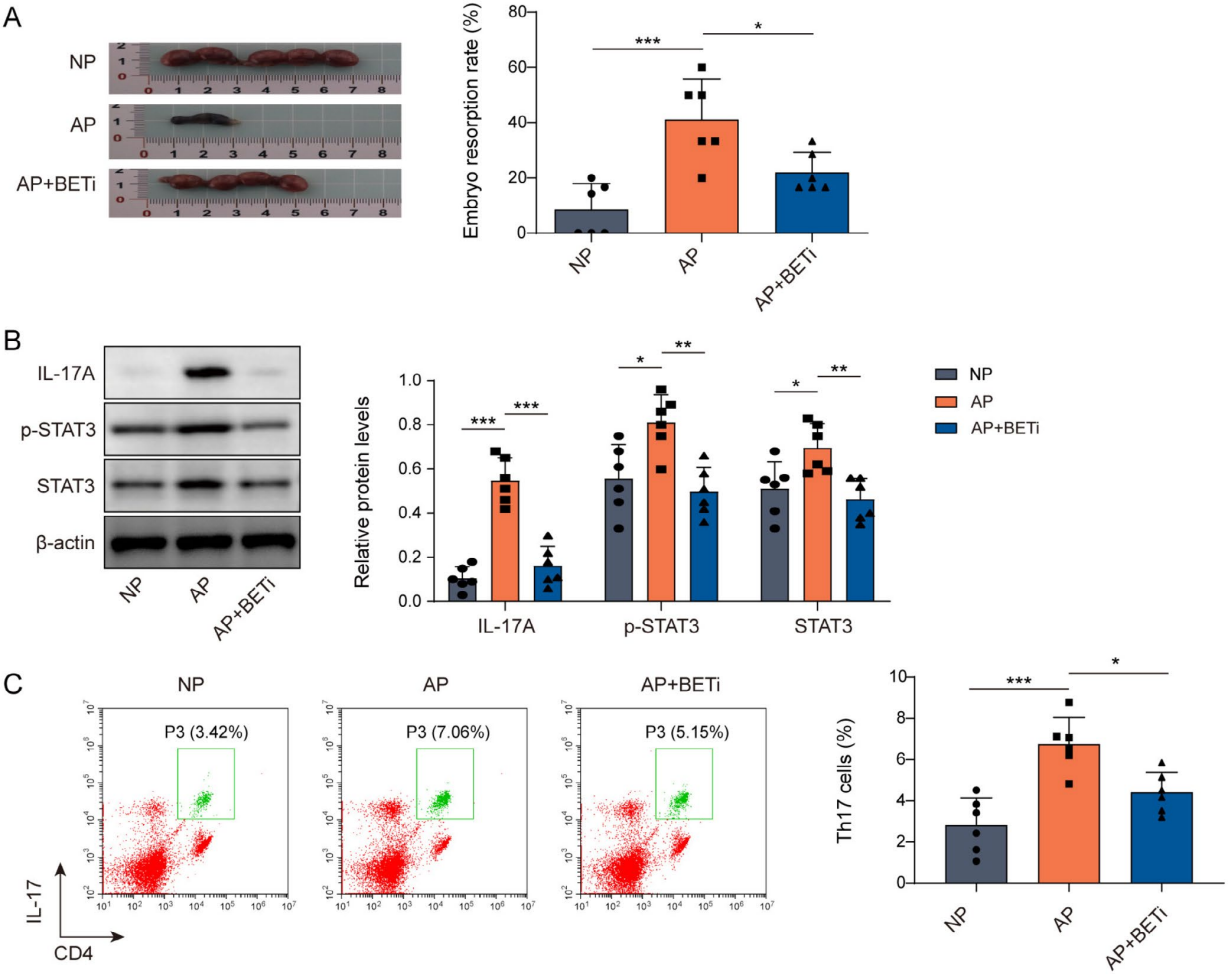
BETi inhibited embryo absorption in miscarried mice by decreasing Th17 cell differentiation. Eight‐week‐old female C57BL/6N and male BALB/C mice were used for crossbreeding, and the crossbred mice were injected intraperitoneally with LPS and MS402 to construct an animal model. (A) Representative macroscopic view of the uterus of each group of mice. The embryo absorption rate of each mouse was determined to evaluate the pregnancy outcome. (B) Western blot was used to detect protein levels of IL‐17A, p‐STAT3, and STAT3 in mouse decidual tissues. (C) Th17 cell differentiation rate was assayed in the peripheral blood of mice by flow cytometry. *n* = 6. *p < 0.05, **p < 0.01, ***p < 0.001.

In the end, we selected 8‐week‐old female C57BL/6N mice crossed with male BALB/C mice to construct the AP animal model, and MS402 was injected intraperitoneally to inhibit BRD4. The results, as shown in Figure 6A, BETi was effective in decreasing the rate of embryo resorption in the AP model mice. In addition, we also found that IL‐17A, p‐STAT3, and STAT3 levels were significantly up‐regulated in the decidual tissues of AP model mice, and BETi reversed the above changes (Figure 6B). Meanwhile, the results in Figure 6C showed that inhibition of BRD4 effectively reduced the differentiation rate of Th17 cells in the peripheral blood of AP mice. The above data suggested that BETi inhibited the STAT3/IL‐17A pathway to reduce Th17 cell differentiation, thus decreasing embryo absorption in miscarried mice.

## 4 Discussion

RSA is a condition with many different etiologies, which affects approximately 1%–2% of women's fertility [37]. However, there is still a lack of effective treatments to alleviate RSA [38]. In the present work, we first reported that BETi suppressed Th17 cell differentiation to mitigate RSA. In addition, this study demonstrated that BETi suppressed the differentiation of Th17 cells by suppressing STAT3‐mediated IL‐17A transcription through inhibition of BRD4. It provides a new therapeutic direction for alleviating RSA in the future.

It was demonstrated that Th17/Treg balance contributed significantly to RSA [39]. The rhCOLI remodels the intra‐maternal immune microenvironment by alleviating the Th17/Treg imbalance, enhancing fertility in RSA mice [40]. Our experiments also revealed that the Th17/Treg ratio was increased in RSA patients. Moreover, promotion of Th17 cell differentiation or reduction of Treg differentiation induced fetal loss [9, 41]. We also found that Th17 differentiation factor levels were elevated in the peripheral blood of RSA patients, while Treg differentiation factor expression was diminished. Moreover, it was reported that BRD4 promoted Th17 cell differentiation [42]. Knockdown of BRD4 significantly reduced IL‐17A expression and inhibited Th17 cell differentiation [43]. Our results indicated that inhibition of BRD4 decreased Th17 cell percentage and the levels of Th17 cell differentiation factors. There are no studies related to BRD4 in RSA, but we discovered that BRD4 was highly expressed in RSA. Besides, energy requirements for Th17 cell differentiation were closely linked to lipid synthesis [44]. The findings of this study suggested that after Th17 cells were induced to differentiate, the expressions of lipid synthesis‐related proteins, ADP/ATP ratio, and NADP/NADPH ratio were increased. However, BETi reversed these results. Our findings first demonstrated that BETi could suppress Th17 cell differentiation to mitigate RSA.

Existing research demonstrated that the expressions of STAT3 and IL‐17A were higher in unexplained RSA patients [26]. We found the levels of STAT3 and IL‐17A were elevated in RSA patients. Previous studies have shown that STAT3 can mediate IL‐17A to play a role in disease. For example, CD28 induces RelA/NF‐κB to activate STAT3, which upregulates IL‐17A expression to promote inflammatory responses [45]. PD‐1 promoted lung fibrosis by increasing STAT3 transcription to up‐regulate IL‐17A and TGF‐β [46]. In addition, STAT3 is intimately connected with Th17 cell differentiation. The palmitoylation cycle promoted Th17 cell differentiation and colitis through activation of STAT3 [47]. Hydrogen sulfide inhibited Th17 cell differentiation by blocking STAT3 phosphorylation, thereby ameliorating systemic lupus erythematosus symptoms in mice [48]. Although it has been shown that STAT3 can directly bind to the IL‐17 promoter to affect Th17 cell differentiation [32, 49], none of the literature seem to have clarified in depth the site where STAT3 acts on the IL‐17 promoter. The present study not only verified that STAT3 promoted Th17 cell differentiation by enhancing IL‐17A transcription but also further clarified the specific site where STAT3 acts with the IL‐17 promoter, which filled the gap of previous studies to a certain extent.

Interestingly, IKK‐α induced NF‐κB transcription to activate STAT3, which combines with BRD4 and recruits it to a subset of STAT3 target genes, thereby inhibiting apoptosis in colorectal cancer [50]. BRD4 promoted liver fibrosis in mice by activating TGFβ1‐induced STAT3 signaling [24]. We found that the BD1 structural domain of BRD4 could combine with STAT3. In addition, BRD4 functions as a transcriptional coactivator that could affect the transcriptional regulation of downstream target genes by transcription factors [20]. At the same time, BRD4 acted by controlling the timing of binding to target gene sites and transcriptional elongation [42]. We experimentally revealed that the inhibition of BRD4 attenuated the interaction of STAT3 with IL‐17A by adding BETi. Furthermore, we found that BETi reduced Th17 cell differentiation and embryonic resorption by down‐regulating STAT3/IL‐17A. In conclusion, this study demonstrated that BETi reduced STAT3 interaction with IL‐17A by inhibiting BRD4. This suggests that BRD4 plays an important role in Th17 differentiation.

Taken together, the collective findings suggest that BETi inhibits STAT3 expression to decrease IL‐17A transcription by targeting BRD4, which hinders Th17 cell differentiation and ultimately alleviates RSA (Graphical abstract). It can be reasonably deduced that the application of BETi may be a promising approach for the treatment of RSA. However, this study is not fully validated in clinical settings despite satisfactory results in animals and cellular models. Furthermore, our results demonstrated significant dysregulation of lipid metabolism in RSA patients relative to the control group. Whether BETi influences RSA occurrence by regulating Th17 cell differentiation and subsequently modulating lipid metabolism remains to be further explored. Clarifying whether BETi affects the differentiation of Th17 cells by inhibiting the STAT3/IL‐17A pathway in patients with RSA is of great significance for formulating precise treatment strategies for RSA.

## Ethics Statement

This study was approved by the ethics committee of The First Hospital of Changsha. All animal experiments were conducted in accordance with the protocol approved by the First Hospital of Changsha Ethics Committee. The ethics code of No. 2024 (08).

## Consent

The informed consent was obtained from study participants.

## Conflicts of Interest

The authors declare no conflicts of interest.

## Supporting information


**Appendix S1:** rmb212682‐sup‐0001‐AppendixS1.docx.

**Appendix S2:** rmb212682‐sup‐0002‐AppendixS2.docx.

**Table S1:** Clinical characteristics of the studied population.

## Data Availability

The Appendices S1 and S2—gating strategy was shown in URL https://osf.io/s9huv/.
