# Supplementary figures and images for: BRD4 Inhibitor Alleviates Recurrent Spontaneous Abortion via Regulating BRD4/STAT3/IL‐17A Axis to Decrease the Th17 Cell Differentiation
Source: Reprod Med Biol. 2025 Oct 15;24(1):e12682. doi: 10.1002/rmb2.12682 (PMC12522068; doi:10.1002/rmb2.12682)

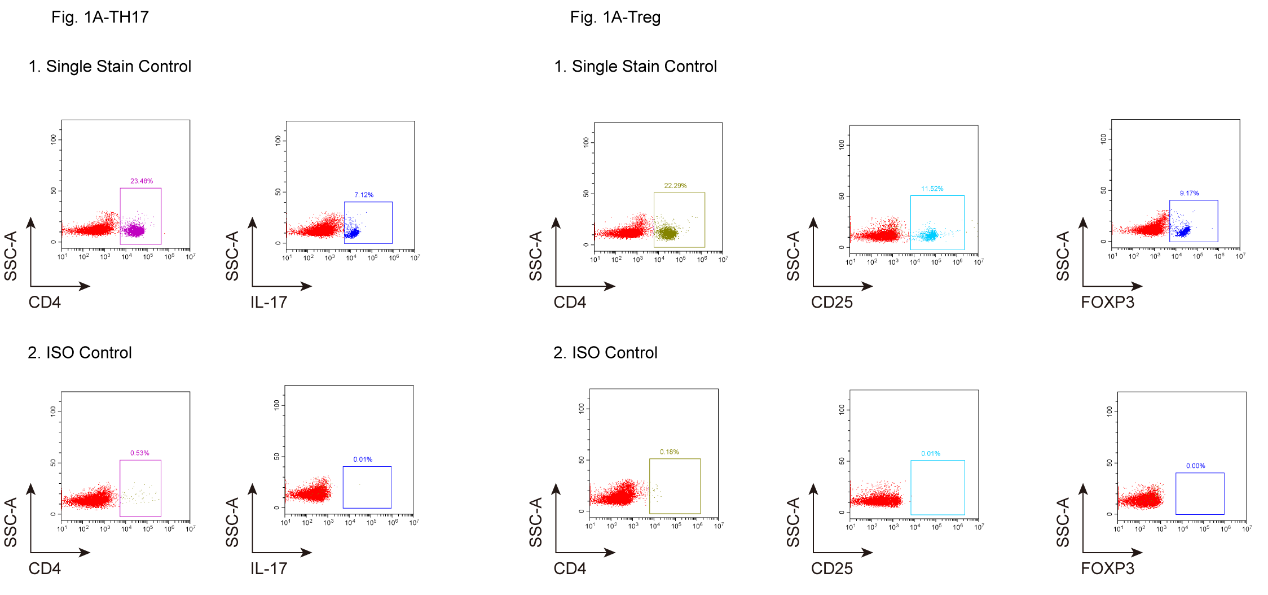


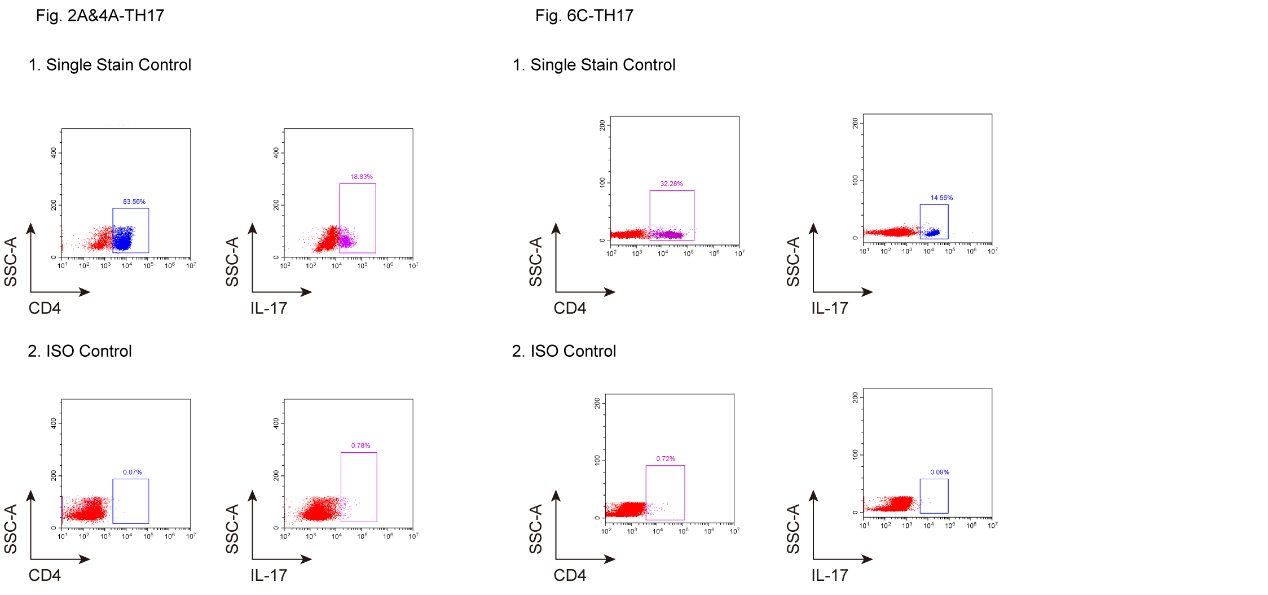

Supplement: Supplementary file 1 — Appendix S1: rmb212682‐sup‐0001‐AppendixS1.docx. [file RMB2-24-e12682-s003.docx]

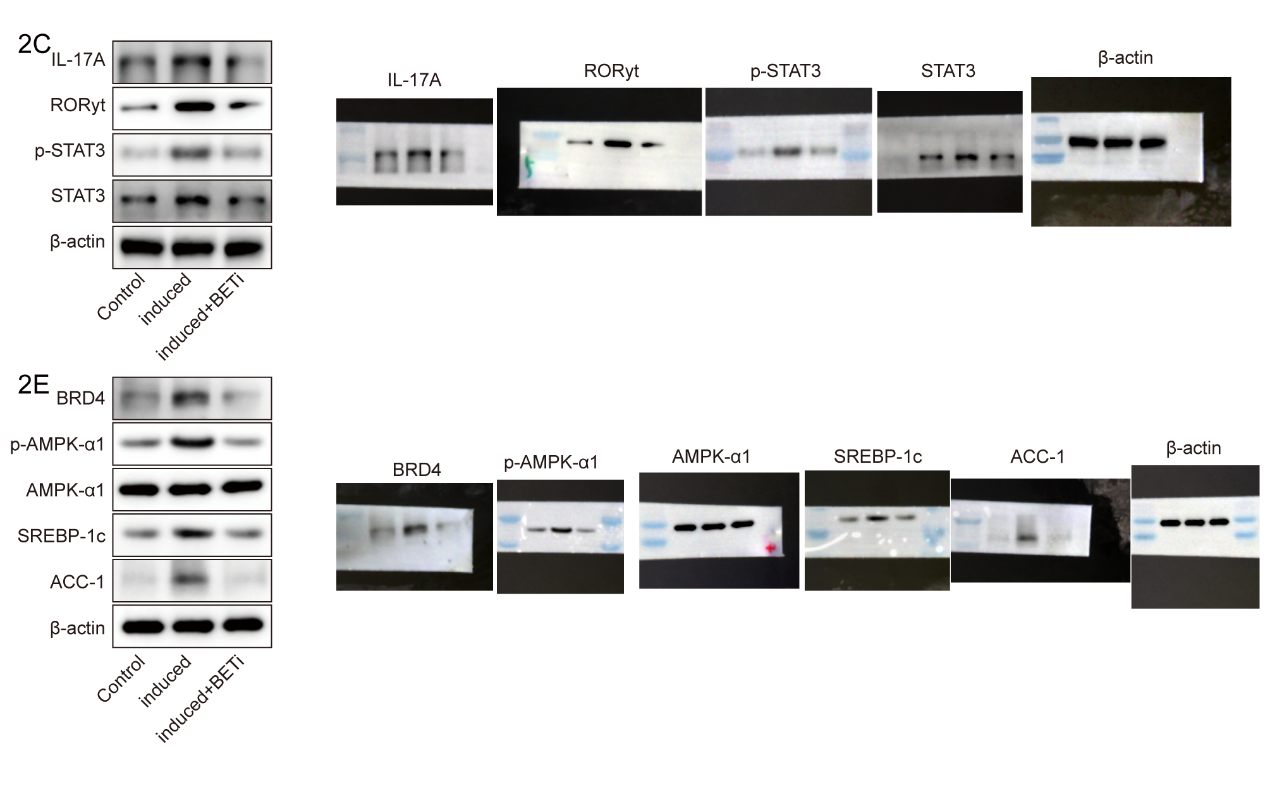

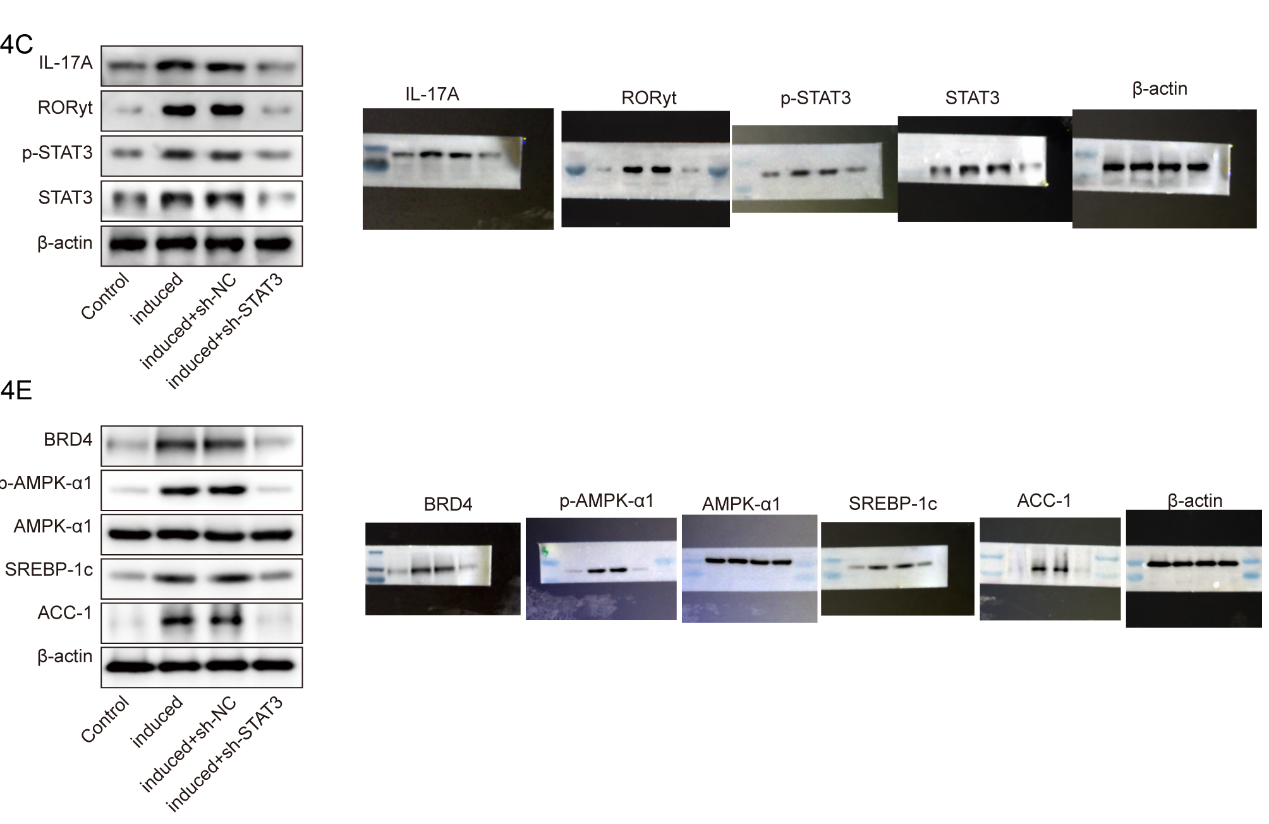


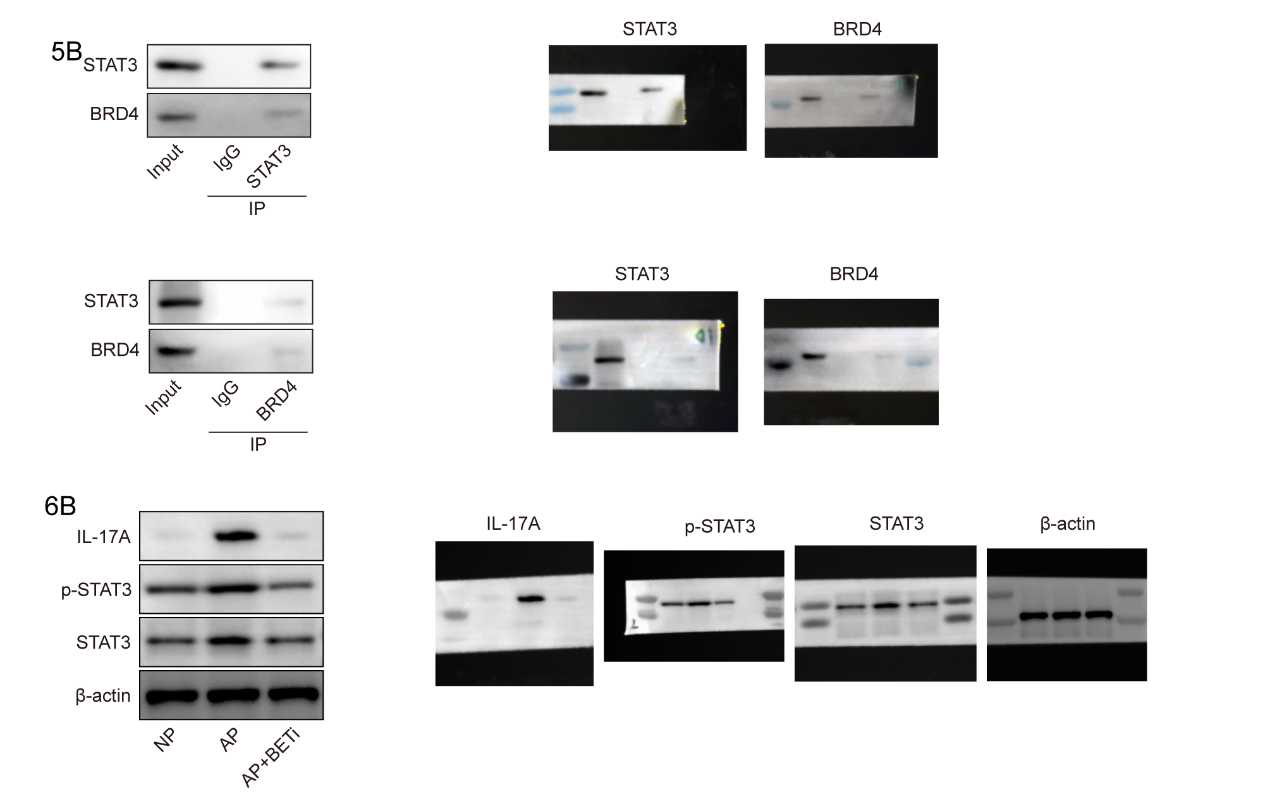

Supplement: Supplementary file 2 — Appendix S2: rmb212682‐sup‐0002‐AppendixS2.docx. [file RMB2-24-e12682-s001.docx]
