# Supplementary material for: BRD4 Inhibitor Alleviates Recurrent Spontaneous Abortion via Regulating BRD4/STAT3/IL‐17A Axis to Decrease the Th17 Cell Differentiation
Source: Reprod Med Biol. 2025 Oct 15;24(1):e12682. doi: 10.1002/rmb2.12682 (PMC12522068; doi:10.1002/rmb2.12682)
Supplement: Supplementary file 3 — Table S1: Clinical characteristics of the studied population. [file RMB2-24-e12682-s002.docx]

**Supplementary Table 1 Clinical characteristics of the studied population**

| **Characteristics** | **NP (Mean±SD) N=15** | **RSA (Mean±SD) N=15** | | ***P* value** |
| --- | --- | --- | --- | --- |
| Maternal age (years) | 26.31±2.11 | 27.74±4.15 | | NS |
| BMI (kg/m2) | 26.8±4.31 | 28.94±3.05 | | NS |
| Systolic blood pressure (mmHg) | 110.74±7.26 | 115.84±8.94 | | NS |
| Diastolic blood pressure (mmHg) | 76.03±4.18 | 77.05±5.22 | | NS |
| Fasting blood pressure (mg/dL) | 92.34±10.06 | 105.31±12.85 | | 0.0046** |
| HDL-Cholesterol (mg/dL) | 40.12±3.62 | 43.62±6.95 | NS | |
| LDL-Cholesterol (mg/dL) | 76.37±10.85 | 95.33±15.17 | 0.0005*** | |
| Triglyceride (mg/dL) | 139.62±25.34 | 188±29.31 | <0.0001*** | |
| Cholesterol (mg/dL) | 140.52±28.12 | 196.37±35.87 | <0.0001*** | |
| Fetal Age (weeks) | 7.53±0.99 | 6.93±1.16 | NS | |
| Primary RM | - | 10 | - | |
| Secondary RM | - | 5 | - | |
| Gestational ages of previous  Miscarriages (weeks±SD) | - | 12.7±2.8 | - | |
| Gravidity | 2.27±0.46 | 4.40±0.91 | <0.0001*** | |
| Number of spontaneous abortion | 0.07±0.26 | 2.8±0.41 | <0.0001*** | |
| Parity | 1.13±0.35 | 0.53±0.52 | 0.0009*** | |

Abbreviations: BMI: Body mass index; HDL: High density lipoprotein; LDL: Low

density lipoprotein; RM: Recurrent miscarriage
